# Metabolic Syndrome without Diabetes or Hypertension Still Necessitates Early Screening for Chronic Kidney Disease: Information from a Chinese National Cross-Sectional Study

**DOI:** 10.1371/journal.pone.0132220

**Published:** 2015-07-10

**Authors:** Daqing Hong, Yuan Zhang, Bixia Gao, Jinwei Wang, Guisen Li, Li Wang, Luxia Zhang, China National Survey of CKD Working Group

**Affiliations:** 1 Division of Nephrology, Sichuan Academy of Medical Sciences & Sichuan Provincial People’s Hospital, Chengdu, China; 2 Peking University Institute of Nephrology, Division of Nephrology, Peking University First Hospital, Beijing, China; The University of Tokyo, JAPAN

## Abstract

Metabolic syndrome (MS) is prevalent, with an increasing contribution to the incidence of chronic kidney disease (CKD). The study of the relationship between them is important. The CKD survey, a national cross-sectional study, provided a large database to accomplish this study. The study population were 41 131 adults from this survey between 2008 and 2009. CKD was defined as estimate glomerular filtration rate (eGFR) less than 60 mL/min per 1.73 m^2^ or the presence of albuminuria. MS was diagnosed by National Cholesterol Education Program—Adult Treatment Panel III (ATPIII), ATPIII-modified or International Diabetes Federation (IDF) criteria. Logistic regression model was applied to study the impact of MS or its components on CKD or its components. The age and sex standardized prevalence of MS by ATPIII, ATPIII-modified and IDF criteria was 11.77% (11.13%–12.40%), 21.51% (20.69%–22.34%) and 16.67% (15.92–17.42)% respectively. Multivariate logistic regression models showed that MS and its components were associated with higher CKD prevalence. The risk for CKD and its components increased with the number of MS components. After adjusting for hypertension and diabetes, the odds ratios of MS for CKD decreased, but remained significantly more than 1 between 1.16(95%CI 1.07–1.26) and 1.37 (95% CI 1.25–1.50) across the different models. Similar results were found with albuminuria, while for decreased eGFR, after adjusting for hypertension and diabetes, the odds ratios of MS and MS components (except elevated TG) became insignificant. In conclusion, MS is prevalent and associated with a higher prevalence of CKD. Different MS components are associated with different risks for CKD, even after adjusting for hypertension and diabetes, which may mainly be contributed more by the increased risk for albuminuria than that for decreased eGFR. More attention must be paid to the population with MS, including those with elevated blood pressure and serum glucose.

## Introduction

Chronic kidney disease (CKD) is a public health problem, with more than 1/10 prevalence in the Chinese population[1]. The leading cause of end stage renal disease in China is different from that reported in Western countries[2, 3]. But with the increasing economic development, the spectrum of diseases is changing, with increase in metabolic disorders[4], an inevitable increased contribution to CKD development, and an increased proportion of Diabetic nephropathy in end-stage renal disease population in China[5]. Previous studies have shown metabolic syndrome (MS) to be a risk factor for CKD[6–10], but the extent to which other components except for diabetes and hypertension contribute to CKD is unclear. Individuals with hypertension and diabetes are more likely to receive clinical intervention, while for the emerging population with metabolic disorders such as elevated blood pressure, elevated serum glucose and overweight are more likely to be ignored.

We did a cross-sectional CKD survey of a nationally representative sample of Chinese adults between September, 2009, and September, 2010. 47 204 participants completed the survey. The adjusted prevalence of eGFR less than 60 mL/min per 1.73 m^2^ was 1.7% (95% CI 1.5–1.9) and of albuminuria was 9.4% (95% CI 8.9–10.0). The overall prevalence of chronic kidney disease was 10.8% (95% CI 10.2–11.3)[1]. With this national wide study, we were able to stratify different impact on CKD by different metabolic disorders. Participants was diagnosed as MS according to ATPIII[11], ATPIII-modified[12] or IDF criteria[13]. Different diagnostic criteria and different adjusted models were applied to precisely identify the population without clinical hypertension or diabetes were still at high risk for CKD.

## Methods

### Data sources/study participants

We used a national CKD survey performed between September, 2009, and September, 2010 using a, multistage, stratified sampling to obtain a representative sample of people aged 18 years or older in the general population from 13 provinces (Beijing, Sichuan, Inner Mongolia Autonomous Region, Jiangsu, Xinjiang Uyghur Autonomous Region, Ningxia Hui Autonomous Region, Zhejiang, Guangxi Zhuang Autonomous Region, Guangdong, Shanghai, Hubei, Hunan, and Shandong). The sampling steps, screening protocol and assessment criteria were reported in detail in a previous report[1]. 50 550 people were invited to participate, of whom 47 204 agreed. Participants with missing values of either of the variables including waist circumference (n = 841), HDL-C (n = 5497), diagnosis of hypertension (n = 243), diagnosis of diabetes (n = 45) or TG (n = 35) were excluded. Finally, 41 131 participants with adequate information were enrolled in this study. The data was obtained in a de-identified and anomymized form.

### Diagnosis criteria

All blood and urine samples were analyzed at the central laboratory in each province, with a successfully completed standardization and certification program among laboratories. Before the study, the central laboratory in each province calibrated creatinine measurements with samples (40 samples with creatinine ranging from 48 μmol/L to 868 μmol/L) at the laboratory of Peking University First Hospital (Beijing, China) to ensure the quality control. Linear regression models from 13 study centers showed that the range of the slopes were 0.95 to 1.12, with the intercepts from -7.7 μmol/L to 3.9 μmol/L. Thus, measurements from local laboratories were used directly for calculation of eGFR.

CKD was defined as eGFR less than 60 mL/min per 1.73 m^2^ or the presence of albuminuria.

eGFR was calculated using an equation developed by adapting of the Modification of Diet in Renal Disease (MDRD) equation on the basis of data from Chinese chronic kidney disease patients[14]. Reduced renal function was defined as an eGFR of less than 60 mL/min per 1.73 m^2^.

$$eGFR=175\times Scr^{-1.234}\times age^{-0.179}[female,\times 0.79]$$

Albuminuria was measured by immunoturbidimetric tests. Urinary creatinine was measured with Jaffe’s kinetic method. The urinary albumin to creatinine ratio (ACR; mg/g creatinine) was calculated. Albuminuria was defined as a urinary albumin to creatinine ratio >30 mg/g creatinine.

Blood pressure was measured by sphygmomanometer three times at 5 minutes intervals. The mean value was calculated for the two closest (if the difference was greater than 10 mm Hg among the readings) or three readings. Hypertension was defined as a systolic blood pressure ≥140 mm Hg, diastolic blood pressure ≥90 mm Hg, any use of antihypertensive medication in the past 2 weeks, or any self-reported history of hypertension.

Fasting blood glucose was measured enzymatically with a glucose oxidase method. Diabetes was defined as fasting plasma glucose ≥7.0 mmol/L, any use of hypoglycaemic agents despite fasting plasma glucose, or any self-reported history of diabetes.

Serum total cholesterol, LDL cholesterol, HDL cholesterol, triglycerides, and uric acid were measured with commercially available reagents. The laboratories used a timed-endpoint colorimetric method to measure LDL cholesterol and HDL cholesterol. Hyperuricaemia was defined by plasma uric acid concentration >420 μmol/L for men and >360 μmol/L for women.

Body height, body weight and waist circumference were measured when the participant was standing and facing directly ahead with his/her shoes and hat off, feet together, and arms by the sides. Body weight was measured with minimal movement and excess clothing removed. Waist circumference was measured 1cm above the umbilicus using an inelastic measuring tape on the bare skin. The tape was circled horizontally around the abdomen without causing compression on the skin when the participant breathed normally with his/her abdomen relaxed. The nearest record of body height, body weight and waist circumference was 0.5cm, 0.5kg and 0.2 cm respectively.

MS was diagnosed according to ATPIII[11], ATPIII-modified[12] or IDF[13] criteria (Table 1).

**Table 1:** Diagnosis Criteria of Metabolic Syndrome.

MS components	ATPIII	ATPIII-modified	IDF
MS	3 or more components	Central obesity plus at least 2 other components	3 or more components
Waist circumference	≥88cm, > = 102cm, if male	≥80cm, > = 90cm, if male	≥80cm, > = 90cm, if male
Blood pressure	SBP≥130 or DBP≥85 mmHg or Taking antihypertensive drugs	SBP≥130 or DBP≥85 mmHg or Taking antihypertensive drugs	SBP≥130 or DBP≥85 mmHg or Taking antihypertensive drugs
Serum glucose level	≥6.1mmol/L or taking diabetes drugs	≥5.6 mmol/L or taking diabetes drugs	≥5.6 mmol/L or taking diabetes drugs
Serum triglyceride level	>1.7 mmol/L	>1.7mmol/L or taking Antilipemic Agents	>1.7mmol/L or taking Antilipemic Agents
HDL-cholesterol	<1.29mmol/L, <1.03mmol/L, if male	<1.29mmol/L, <1.03mmol/L, if male	<1.29mmol/L, <1.03mmol/L, if male

### Statistical analysis

The prevalence estimates were weighted by China Population Sampling Census in 2009 data to represent the total adult population in China.

Continuous data are presented as means with SDs or medians (IQR). Categorical variables are presented as proportions. Characteristics are described and stratified by the presence or absence of MS according to different criteria. Prevalence of MS and its components are presented and stratified by gender.

Demographic characteristics, history of diseases and medications, physical examinations, and laboratory findings were compared between MS-present and MS-absent groups according to different MS diagnosis criteria. Continuous data were compared between two groups using *t-test* or non-parametric Wilcoxon rank sum test for severely skewed data. Chi-square test was used to compare categorical variables between two groups. Binary logistic regression was used to calculate the crude and adjusted model for CKD and its components by MS, its components and the number of the components. 5 adjusted models were used, including: MODEL1: adjustment for age, sex, hypertension and diabetes. MODEL2: MODEL1 plus adjustment for history of cardiovascular disease (myocardial infarction or stroke); MODEL3: MODEL2 plus adjustment for former kidney disease and nephrotoxic drugs (somedon, APC [both analgesic mixtures containing phenacetin] ibuprofen, and herbal pills containing aristolochic acid [Long Dan Xie Gan, Guan Xin Su He, and Pai Shi Ke Li]). MODEL4: MODEL3 plus adjustment for hyperuricaemia. MODEL5: MODEL4 plus adjustment for smoking, alcohol, regular exercise, education and income. The ORs for CKD by different compositions of MS components (IDF or ATPIII-modified criteria) were calculated with adjustment for age and gender. The p value for trend was obtained by directly including the number of components as an ordinal categorical variable in the regression model.

All analyses were performed with SAS, version 9.1.3 (SAS Institute Inc). A p value of less than 0.05 was considered significant. The study protocol was approved by the ethics committee of Peking University First Hospital. All participants gave written informed consent before data collection.

## Results

### Comparison of population characteristics (Table 2)

50 550 people were invited to participate, of whom 47 204 completed the survey and examination. 41 131 participants with adequate information were enrolled. Table 1 shows the characteristics of the participants with or without MS according to the various criteria, the average age was 50±15 years, with a male proportion of 41.91%. The standardized prevalence of MS according to ATPIII, ATPIII-modified or IDF criteria was 11.77% (11.13–12.40), 21.51% (20.69–22.34) and 16.67% (15.92–17.42) respectively (S1 Table). Only 25.42% (24.45–26.38) of participants did not have any MS components.

**Table 2:** Characteristics of Participants.

Variables		MS(ATP-III)		MS(ATP-III)-modified		MS(IDF)	
	All	absent	present	absent	present	absent	present
N	41131	34918	6213	29993	11138	32016	9115
Age(yrs)	50±15	49±15	58±13*	48±15	57±13*	48±15	57±13*
Male (%)	41.91	43.45	33.30*	43.36	38.01*	43.88	34.57*
Educational status (high school and above) (%)	42.95	44.33	35.16*	44.66	38.33*	44.49	37.56*
Have medical insurance (%)	83.87	83.44	86.27*	82.78	86.80*	83.13	86.45*
Rural residents (%)	46.90	47.71	42.35*	48.79	41.79*	48.18	42.39*
Cardio-cerebral-vascular disease (%)	2.47	1.99	5.13*	1.51	5.05*	1.81	4.78*
Hypertension (%)	33.54	26.65	72.23*	21.52	65.91*	24.35	65.82*
Diabetes (%)	7.46	3.61	29.05*	2.60	20.52*	4.18	18.98*
HBV infection (%)	2.69	2.80	2.08*	2.84	2.29*	2.84	2.16*
History of nephrotoxic drugs (%)	3.25	3.03	4.47*	2.85	4.33*	2.93	4.38*
Former history of kidney disease (%)	4.91	4.68	6.16*	4.49	6.02*	4.59	6.02*
Current smoking (%)	23.97	24.46	21.21*	24.38	22.86*	24.61	21.72*
Current drinking (%)	24.50	25.12	21.00*	24.66	24.06	24.74	23.64^#^
Creatinine (mmol/L)	74.7±20.7	74.4±20.6	75.9±21.1*	74.1±20.7	76.2±20.6*	74.3±20.7	75.7±20.4*
eGFR (ml/min/1.73m^2^)	101.0±27.5	102.1±27.6	94.6±25.5*	103.1±27.9	95.2±25.4*	102.6±27.9	95.1±25.2*
Decreased eGFR (%)	2.56	2.18	4.73*	1.94	4.24*	2.12	4.10*
ACR (mg/g creatinine; median [IQR])	7.2 (3.9–14.5)	6.8 (3.7–13.4)	10.5 (5.5–22.6) *	6.6 (3.6–12.8)	9.4 (5.0–20.0) *	6.7 (3.6–13.2)	9.5 (5.1–20.2) *
Albuminuria (%)	7.75	6.46	14.95*	5.85	12.85*	6.29	12.86*
Microalbuminuria (%)	7.05	5.98	13.02*	5.44	11.38	5.81	11.40
Macroalbuminuria (%)	0.70	0.48	1.93*	0.41	1.47	0.48	1.46
CKD (%)	9.74	8.18	18.46*	7.45	15.91*	8.01	15.82*
Plasma uric acid (umol/L)	302.4±93.8	298.2±91.1	331.4±106.0*	294.3±89.0	329.7±103.8*	297.1±90.8	326.8±103.0*
Hyperuricaemia (%)	10.43	9.54	15.44*	8.84	14.72*	9.46	13.81*
WC (cm)	80.8±11.3	79.0±9.6	91.0±14.5*	77.5±9.1	89.5±12.0*	77.7±9.0	91.8±11.8*
Triglyceride (mmol/L)	1.11 (0.76–1.69)	1.01 (0.71–1.45)	2.17 (1.71–2.99)*	0.96 (0.69–1.34)	1.87 (1.89–2.63)*	0.99 (0.70–1.43)	1.83 (1.24–2.57)*
HDL-C(mmol/L)	1.41±0.39	1.46±0.38	1.13±0.29*	1.48±0.38	1.20±0.32*	1.46±0.39	1.21±0.32*
LDL-C(mmol/L)	2.95±0.95	2.89±0.93	3.24±1.00*	2.84±0.91	3.24±1.00*	2.86±0.92	3.25±0.99
Cholesterol (mmol/L)	4.91±1.33	4.83±1.29	5.30±1.49*	4.78±1.28	5.24±1.42*	4.82±1.32	5.24±1.34*
BMI (kg/m^2^)	23.9±3.7	23.3±3.3	27.3±3.7*	22.9±3.2	26.7±3.4*	22.9±3.1	27.4±3.2*
Obesity (BMI±25 kg/m^2^)	35.71	29.07	72.99*	23.01	69.90*	23.46	78.72*
Systolic blood pressure (mmHg)	127.±21	124.2±19.6	143.2±19.7*	122.0±18.6	140.7±20.2*	123.2±19.1	141.0±20.5*
Diastolic blood pressure (mmHg)	80±12	78.7±11.2	88.3±11.1*	77.5±10.8	87.3±11.2*	78.1±11.0	87.5±11.4*

*Note*: *: *P<0*.*001* between MS present and MS absent groups; #: *P<0*.*05* between MS present and MS absent groups.

The prevalence and the component number of MS were higher in the female population (S1 Table). Participants with MS were older, with lower education, wider insurance coverage, more rural, more history of cardio-cerebral-vascular disease, less HBV-infected rate, more history of nephrotoxic drugs, more history of former kidney disease, less current smokers, and less current drinkers than participants without MS. The average level of parameters of MS components and the prevalence of MS components were higher in the MS present group than the MS absent group.

### Differences of CKD or its components between MS-present and MS-absent groups

Table 2 shows serum creatinine was higher, eGFR was lower and the prevalence of decreased eGFR was higher in the MS-present group, and that urine ACR, and the prevalence of albuminuria was higher in the MS-present group. The prevalence of CKD was higher in the MS-present population (*P*<0.001). S2 Table shows the prevalence of CKD or its components was higher in the population with MS or with MS components.

### Impact on CKD by MS or MS components

Table 3 presents the ORs for CKD by MS, MS components and numbers of MS components in different models. After adjustment for age and sex, presence of MS (different criteria, ORs between 1.69[95%CI 1.57–1.81] and 1.96[95%CI 1.82–2.12]) or presence of any of the MS components was associated with higher prevalence for CKD, and the ORs increased with increasing numbers of MS components (*P* for trend *<0*.*0001*). After adjustment for diabetes and hypertension, the ORs of MS decreased dramatically, but were still significantly greater than 1, between 1.16(95%CI 1.07–1.26) and 1.37 (95% CI 1.25–1.50) across the different models.

**Table 3:** Association between Metabolic Syndrome and Chronic Kidney Disease.

Participants (n = 41131)	Crude OR (95% confidence interval)	Age and sex adjusted OR (95% confidence interval)	Multivariable adjusted OR1 (95% confidence interval)	Multivariable adjusted OR2 (95% confidence interval)	Multivariable adjusted OR3 (95% confidence interval)	Multivariable adjusted OR4 (95% confidence interval)	Multivariable adjusted OR5 (95% confidence interval)
Cenral OB1	2.02(1.86–2.20)	1.56(1.43–1.71)	1.21(1.11–1.33)	1.21(1.10–1.32)	1.20(1.10–1.32)	1.33(1.22–1.46)	1.31(1.19–1.43)
Cenral OB2	1.74(1.63–1.86)	1.43(1.33–1.53)	1.14(1.06–1.22)	1.14(1.06–1.22)	1.13(1.05–1.21)	1.25(1.16–1.34)	1.24(1.15–1.33)
OB (BMI)	1.45(1.36–1.55)	1.28(1.19–1.36)	0.996(0.928–1.069)	0.99(0.93–1.07)	0.99(0.92–1.06)	1.10(1.02–1.18)	1.10(1.02–1.18)
Low HDL-C	1.34(1.25–1.43)	1.31(1.22–1.40)	1.17(1.09–1.26)	1.16(1.08–1.25)	1.16(1.08–1.25)	1.21(1.12–1.30)	1.18(1.10–1.28)
High BP	2.97(2.77–3.19)	2.23(2.07–2.41)	1.32(1.18–1.48)	1.32(1.18–1.48)	1.32(1.17–1.48)	1.36(1.21–1.52)	1.34(1.20–1.51)
High FPG	2.05(1.91–2.19)	1.56(1.45–1.67)	1.08(0.99–1.18)	1.087(0.995–1.188)	1.09(1.000–1.194)	1.15(1.05–1.26)	1.18(1.08–1.30)
High TG	1.56(1.46–1.67)	1.37(1.28–1.46)	1.13(1.06–1.22)	1.13(1.06–1.22)	1.12(1.04–1.20)	1.18(1.10–1.27)	1.22(1.13–1.31)
1 component	1.93(1.71–2.18)	1.61(1.42–1.82)	1.33(1.17–1.50)	1.33(1.17–1.51)	1.32(1.16–1.50)	1.33(1.17–1.51)	1.32(1.16–1.50)
2 components	2.78(2.47–3.13)	2.05(1.82–2.32)	1.44(1.42–1.87)	1.44(1.27–1.64)	1.43(1.26–1.63)	1.49(1.30–1.69)	1.48(1.30–1.69)
3 components	3.90(3.45–4.40)	2.67(2.35–3.02)	1.63(1.42–1.87)	1.63(1.42–1.87)	1.61(1.41–1.85)	1.76(1.54–2.03)	1.76(1.53–2.02)
4 components	4.73(4.14–5.40)	3.10(2.71–3.56)	1.64(1.41–1.92)	1.63(1.40–1.91)	1.61(1.38–1.88)	1.89(1.61–2.20)	1.91(1.63–2.23)
5 components	6.15(5.18–7.31)	3.86(3.24–4.61)	1.68(1.37–2.04)	1.66(1.36–2.03)	1.63(1.33–1.99)	2.05(1.68–2.51)	2.06(1.68–2.52)
Trend across number of components	*P<0*.*0001*	*P<0*.*0001*	*P<0*.*0001*	*P<0*.*0001*	*P<0*.*0001*	*P<0*.*0001*	*P<0*.*0001*
MS (ATP-III)	2.54(2.36–2.74)	1.96(1.82–2.12)	1.27(1.17–1.39)	1.27(1.16–1.38)	1.26(1.16–1.38)	1.37(1.25–1.50)	1.37(1.25–1.49)
MS (IDF)	2.16(2.02–2.31)	1.69(1.57–1.81)	1.17(1.08–1.27)	1.17(1.08–1.26)	1.16(1.07–1.26)	1.31(1.21–1.41)	1.31(1.21–1.41)
MS (ATP-III modified)	2.35(2.20–2.51)	1.82(1.70–1.95)	1.22(1.13–1.32)	1.21(1.12–1.31)	1.21(1.12–1.31)	1.33(1.23–1.44)	1.34(1.24–1.45)

OR1: adjustment for age, sex, hypertension and diabetes. OR2: OR1 plus adjustment for cardiovascular disease; OR3: OR2 plus adjustment for former kidney disease and nephrotoxic drugs. OR4: OR3 plus adjustment for hyperuricaemia. OR5: OR4 plus adjustment for smoking, alcohol, regular exercise, and income.

Central OB1: obesity according to WC by ATP-III; Central OB2: obesity according to WC by ATP-III modified or IDF; Obesity (BMI): > = 25Kg/m^2^, only obesity by IDF or ATP-III modified criteria was used to calculate MS components.

### Relationship between CKD and different compositions of MS components

**Fig 1:**
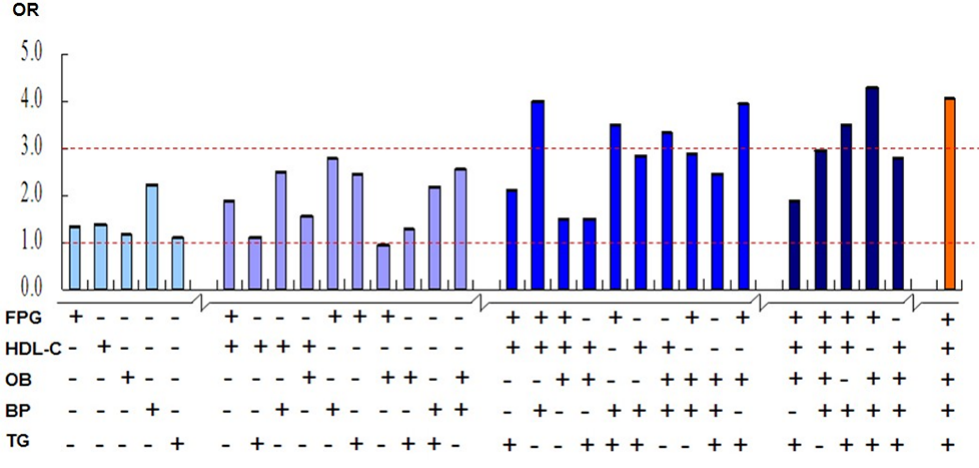
OR for CKD by different compositions of MS components compared to participants without any of MS components. OR was calculated by logistic regressions adjusted for age and gender, obesity according to IDF or ATP-III was used in the analysis. *y*-axis represent OR for CKD, *x*-axis represent the compositions of MS components. ‘+’ indicated presence of the component at the same line and ‘-’ indicated absence of component at the same line. Different colors of vertical bars represent different numbers of MS components.

Fig 1 shows the ORs for CKD increasing with increasing number of MS components. High blood pressure and high fasting plasma glucose (FPG) presented more frequently in compositions of MS components with ORs>3 for CKD.

### Impact on decreased eGFR by MS or MS components

Table 4 presents the ORs for decreased eGFR by MS, MS components and numbers of MS components in different models. After adjustment for age and sex, presence of MS (different criteria, ORs between 1.29[95%CI 1.12–1.48] and 1.42[95%CI 1.23–1.64]) and presented components except obesity, were associated with greater prevalence of decreased eGFR, and ORs increased with the increasing numbers of MS components (trend *P<0*.*0001*). After adjusting for diabetes and hypertension, the ORs decreased dramatically, the relationship between MS and decreased eGFR became insignificant. ORs of MS components, except for elevated TG for decreased eGFR (ORs between 1.36[95% CI 1.20–1.55] and 1.46[95% CI 1.28–1.66]), became statistically insignificant. ORs for decreased eGFR increased with the number of MS components even after adjusting for hypertension and diabetes (*P* for trend = 0.0293), while the significance was not present in all of the confounder adjusted models.

**Table 4:** Association between Metabolic Syndrome and Decreased eGFR.

Participants (n = 41131)	Crude OR (95% confidence interval)	Age and sex adjusted OR (95% confidence interval)	Multivariable adjusted OR1 (95% confidence interval)	Multivariable adjusted OR2 (95% confidence interval)	Multivariable adjusted OR3 (95% confidence interval)	Multivariable adjusted OR4 (95% confidence interval)	Multivariable adjusted OR5 (95% confidence interval)
Cenral OB1	1.81(1.55–2.11)	1.14(0.97–1.35)	0.99(0.84–1.17)	0.997(0.843–1.179)	0.99(0.83–1.17)	1.03(0.87–1.22)	1.004(0.847–1.191)
Cenral OB2	1.57(1.39–1.77)	1.13(0.99–1.28)	0.98(0.86–1.12)	0.988(0.865–1.128)	0.98(0.86–1.12)	1.01(0.89–1.16)	1.01(0.88–1.15)
OB (BMI)	1.23(1.08–1.39)	1.03(0.91–1.17)	0.90(0.79–1.02)	0.90(0.789–1.026)	0.88(0.77–1.01)	0.92(0.80–1.05)	0.91(0.80–1.04)
Low HDL-C	1.21(1.06–1.38)	1.18(1.03–1.35)	1.10(0.96–1.26)	1.11(0.97–1.27)	1.09(0.95–1.26)	1.11(0.96–1.27)	1.08(0.94–1.24)
High BP	2.77(2.43–3.17)	1.43(1.24–1.65)	1.01(0.81–1.26)	1.02(0.82–1.26)	1.01(0.81–1.26)	1.02(0.82–1.27)	1.01(0.81–1.25)
High FPG	2.03(1.79–2.30)	1.25(1.10–1.43)	0.89(0.75–1.05)	0.88(0.75–1.04)	0.89(0.75–1.06)	0.91(0.77–1.08)	0.94(0.79–1.11)
High TG	1.91(1.69–2.16)	1.56(1.38–1.77)	1.40(1.23–1.60)	1.40(1.23–1.59)	1.36(1.20–1.55)	1.39(1.22–1.59)	1.46(1.28–1.66)
1 component	2.54(1.97–3.28)	1.68(1.30–2.18)	1.53(1.18–2.00)	1.53(1.18–1.99)	1.51(1.16–1.97)	1.51(1.16–1.97)	1.51(1.16–1.97)
2 components	3.78(2.95–4.85)	1.99(1.55–2.57)	1.66(1.27–2.17)	1.66(1.27–2.17)	1.64(1.25–2.14)	1.66(1.27–2.17)	1.66(1.27–2.17)
3 components	5.11(3.97–6.56)	2.35(1.81–3.04)	1.79(1.35–2.37)	1.79(1.35–2.36)	1.75(1.32–2.31)	1.81(1.36–2.39)	1.81(1.37–2.40)
4 components	5.51(4.20–7.24)	2.36(1.78–3.12)	1.63(1.20–2.22)	1.64(1.20–2.32)	1.58(1.16–2.16)	1.67(1.22–2.28)	1.71(1.25–2.34)
5 components	6.37(4.52–8.97)	2.51(1.77–3.57)	1.51(1.03–2.23)	1.53(1.03–2.25)	1.44(0.97–2.13)	1.56(1.05–2.32)	1.58(1.07–2.35)
Trend across number of components	*P<0*.*0001*	*P<0*.*0001*	*P = 0*.*0293*	*P = 0*.*0249*	*P = 0*.*0531*	*P = 0*.*0123*	*P = 0*.*0075*
MS (ATP-III)	2.23(1.95–2.56)	1.42(1.23–1.64)	1.07(0.91–1.25)	1.07(0.91–1.26)	1.06(0.90–1.24)	1.08(0.92–1.28)	1.08(0.92–1.27)
MS (IDF)	1.97(1.73–2.24)	1.29(1.12–1.48)	1.03(0.89–1.19)	1.04(0.90–1.20)	1.02(0.88–1.18)	1.06(0.92–1.23)	1.07(0.92–1.24)
MS (ATP-III modified)	2.24(1.98–2.53)	1.42(1.25–1.62)	1.11(0.96–1.28)	1.11(0.97–1.29)	1.10(0.95–1.27)	1.14(0.98–1.31)	1.15(0.99–1.33)

OR1: adjustment for age, sex, hypertension and diabetes. OR2: OR1 plus adjustment for cardiovascular disease; OR3: OR2 plus adjustment for former kidney disease and nephrotoxic drugs. OR4: OR3 plus adjustment for hyperuricaemia. OR5: OR4 plus adjustment for smoking, alcohol, regular exercise, and income.

Central OB1: obesity according to WC by ATP-III; Central OB2: obesity according to WC by ATP-III modified or IDF; Obesity (BMI): > = 25Kg/m^2^, only obesity by IDF or ATP-III modified criteria was used to calculate MS components.

### Impact on albuminuria by MS or MS components

Table 5 presents the ORs for albuminuria by MS, MS components and numbers of MS components in different models. After adjustment for age and sex, presence of MS (different criteria, ORs between 1.82[95%CI 1.68–1.97] and 2.09[95%CI 1.92–2.27]) and presence of any of the MS(ORs between 1.30[1.21–1.41]and 2.58[2.37–2.81]) was associated with greater prevalence for albuminuria. The ORs increased with the increasing number of MS components (*P* for trend *<0*.*0001*). After adjustment for diabetes and hypertension, the ORs decreased dramatically, while still greater than 1 between 1.21(1.11–1.32) and 1.42(1.29–1.57) of MS, and between 1.17 (95%CI 1.08–1.27) and 1.52(95%CI 1.34–1.73) of MS components (except for high TG) across the different models.

**Table 5:** Association between metabolic syndrome and albuminuria.

Participants (n = 41131)	Crude OR (95% confidence interval)	Age and sex adjusted OR (95% confidence interval)	Multivariable adjusted OR1 (95% confidence interval)	Multivariable adjusted OR2 (95% confidence interval)	Multivariable adjusted OR3 (95% confidence interval)	Multivariable adjusted OR4 (95% confidence interval)	Multivariable adjusted OR5 (95% confidence interval)
Cenral OB1	2.03(1.86–2.22)	1.68(1.52–1.85)	1.26(1.14–1.39)	1.26(1.14–1.39)	1.25(1.31–1.38)	1.41(1.27–1.56)	1.38(1.25–1.53)
Cenral OB2	1.78(1.66–1.92)	1.54(1.42–1.66)	1.19(1.10–1.29)	1.19(1.10–1.28)	1.18(1.09–1.28)	1.33(1.22–1.44)	1.32(1.22–1.43)
OB (BMI)	1.51(1.40–1.63)	1.36(1.27–1.47)	1.04(0.96–1.12)	1.03(0.95–1.11)	1.02(0.95–1.11)	1.16(1.07–1.26)	1.16(1.07–1.26)
Low HDL-C	1.37(1.27–1.48)	1.34(1.24–1.45)	1.19(1.10–1.29)	1.18(1.09–1.27)	1.17(1.08–1.27)	1.23(1.14–1.34)	1.21(1.12–1.32)
High BP	3.07(2.83–3.32)	2.58(2.37–2.81)	1.47(1.30–1.67)	1.47(1.29–1.67)	1.47(1.29–1.67)	1.52(1.34–1.73)	1.51(1.33–1.71)
High FPG	2.07(1.92–2.23)	1.69(1.56–1.83)	1.19(1.08–1.31)	1.19(1.08–1.31)	1.20(1.09–1.32)	1.27(1.15–1.40)	1.31(1.19–1.44)
High TG	1.45(1.34–1.56)	1.30(1.21–1.41)	1.06(0.98–1.14)	1.05(0.97–1.14)	1.04(0.96–1.13)	1.11(1.03–1.20)	1.13(1.05–1.23)
1 component	1.82(1.59–2.08)	1.61(1.40–1.84)	1.29(1.13–1.49)	1.30(1.13–1.49)	1.29(1.12–1.48)	1.30(1.13–1.49)	1.29(1.12–1.49)
2 components	2.61(2.29–2.98)	2.13(1.86–2.43)	1.43(1.24–1.66)	1.43(1.24–1.65)	1.42(1.23–1.65)	1.48(1.28–1.71)	1.47(1.28–1.70)
3 components	3.68(3.22–4.21)	2.85(2.48–3.27)	1.65(1.41–1.92)	1.64(1.41–1.91)	1.63(1.40–1.90)	1.80(1.54–2.10)	1.80(1.54–2.09)
4 components	4.58(3.96–5.29)	3.43(2.95–3.99)	1.70(1.44–2.02)	1.69(1.42–2.00)	1.67(1.41–1.98)	2.00(1.68–2.37)	2.01(1.70–2.39)
5 components	6.19(5.14–7.45)	4.50(3.72–5.44)	1.82(1.47–2.26)	1.80(1.45–2.23)	1.76(1.42–2.19)	2.31(1.86–2.88)	2.31(1.86–2.88)
Trend across number of components	*P<0*.*0001*	*P<0*.*0001*	*P<0*.*0001*	*P<0*.*0001*	*P<0*.*0001*	*P<0*.*0001*	*P<0*.*0001*
MS (ATP-III)	2.55(2.35–2.76)	2.09(1.92–2.27)	1.31(1.19–1.44)	1.30(1.18–1.43)	1.29(1.17–1.42)	1.42(1.29–1.57)	1.42(1.28–1.56)
MS (IDF)	2.20(2.04–2.37)	1.82(1.68–1.97)	1.22(1.12–1.33)	1.22(1.12–1.32)	1.21(1.11–1.32)	1.38(1.27–1.51)	1.38(1.27–1.51)
MS (ATP-III modified)	2.37(2.20–2.55)	1.96(1.81–2.11)	1.26(1.16–1.37)	1.26(1.15–1.37)	1.25(1.15–1.36)	1.40(1.28–1.53)	1.41(1.29–1.54)

*Note*: OR1: adjustment for age, sex, hypertension and diabetes. OR2: OR1 plus adjustment for cardiovascular disease; OR3: OR2 plus adjustment for former kidney disease and nephrotoxic drugs. OR4: OR3 plus adjustment for hyperuricaemia. OR5: OR4 plus adjustment for smoking, alcohol, regular exercise, and income.

Central OB1: obesity according to WC by ATP-III; Central OB2: obesity according to WC by ATP-III modified or IDF; Obesity (BMI): > = 25Kg/m^2^, only obesity by IDF or ATP-III modified criteria was used to calculate MS components.

## Discussion

Chronic kidney disease is a public health problem with many risk factors and its progression is hard to control. Thus it is more important to prevent the occurrence of this disease rather than to manage it. Metabolic syndrome was reported to be a risk factor for CKD. In this national wide survey we found that MS, according to any set of diagnostic criteria, was associated with a higher CKD prevalence in China. MS was shown to be associated with higher risk for CKD. The ORs for CKD increased with the increasing number of MS components in agreement with previous reports[6–8, 15, 16]. Patients with diabetes or hypertension are more likely to have medical intervention, although there are still a large proportion of patients with latent metabolic disorders that are frequently ignored. Thus some studies have adjusted diabetes and hypertension, and found that patients with MS are still at higher risk for CKD and CKD progression. This population may necessitate more attention than before, especially for screening and prevention. One study showed that MS, even adjusting for diabetes and hypertension, was associated with higher risk for CKD in a southern Chinese population[7]. These results were further confirmed in our national cross-sectional study. The ORs decreased dramatically after adjustment for hypertension and diabetes. And among the compositions of MS components, hypertension and diabetes presented more frequently in the compositions with ORs greater than 3 for CKD. These findings can be interpreted as follows: hypertension and diabetes is still the leading issue of MS[17, 18] to which we should pay more attention in the prevention of onset of CKD, while the MS-present population without diabetes and hypertension still need special care[9, 19, 20], because they are more likely to be ignored.

We also found a positive relationship between the prevalence of MS or its components and the presence of CKD injury indices, including decreased eGFR and albuminuria. In this study we compared different impacts on the prevalence of decreased eGFR and albuminuria by MS and its components.

Albuminuria was recognized as an indicator of endothelial damage. In this study, presence of any of the MS components was associated with increased ORs for albuminuria, and the ORs increased with the increasing number of MS components. This relationship between MS or MS components and albuminuria remained significant after adjusting for diabetes or hypertension. This is in accordance with most studies[16, 21–23]. In a Japanese study[21], metabolic syndrome was reported to be associated with the development of proteinuria in subjects (HR, 1.67), even in those without hypertension, diabetes, or cardiovascular disease (HR, 1.64). Besides diabetes, insulin resistance was also reported to be associated with increased risk for albuminuria[24]. In addition, decreases in waist circumference was reported to be associated with reductions in urinary albumin excretion in the PREMIER study [25]. Our study showed that the prevalence of albuminuria accounted for more than 80% of the prevalence of CKD. Considering the intimate relationship between MS and albuminuria, population with MS components are the target population for CKD screening, despite the absence of hypertension and diabetes.

In regard to decreased eGFR, the presence of MS components except obesity was associated with higher risk in the age-and-sex-adjusted model, while the significance disappeared after adjusting for diabetes and hypertension except for high TG. This suggests that diabetes and hypertension rather than other MS components may be more intimately associated with GFR loss. Previous studies present different results: Some have reported a positive association between decreased GFR and metabolic syndrome independent of diabetes and/or hypertension. In the Atherosclerosis Risk in Communities study[9], after adjusting for the subsequent development of diabetes and hypertension during the follow-up, the OR of incident decreased eGFR among participants with the metabolic syndrome was 1.24 (95% CI, 1.01 to 1.51). In Asia, a prospective cohort study in Korea [19] in 10 685 healthy men without CKD, after adjustment for age, baseline GFR, and uric acid level, MS at baseline was associated with a significantly increased risk of decreased eGFR (HR, 1.99; 95% confidence interval, 1.46 to 2.73). In Japan, a prospective observational cohort study (Niigata Preventive Medicine Study), which included 34 986 participants without baseline kidney disease, was based upon annual health examinations [21]. The metabolic syndrome was associated with development of kidney dysfunction (hazard ratio [26], 2.12). The association of metabolic syndrome with kidney dysfunction remained significant in subjects without hypertension, diabetes, or cardiovascular disease (HR, 1.99). A positive association between MS and decreased GFR is found in the above studies, while the risk for developing decrease GFR become lower after adjustment for diabetes and/or hypertension. Other studies have reported that after exclusion of the impact by diabetes and hypertension, the significance of MS for decreased GFR disappeared. 380 obese non-diabetic subjects were studied in Italy [27]. Subjects with MS had lower eGFR and greater prevalence of CKD (decreased eGFR), but the differences of eGFR between patients with and without MS were no longer statistically significant after adjusting for age (*P =* 0.99). Similarly, after adjusting for age, individuals affected by MS did not show a significantly increased risk of CKD (OR = 1.2, 95% CI = 0.5–2.8, *P =* 0.94) as compared to patients who were not affected by MS. In a prospective study Tehran Lipid and Glucose Study[17], individuals with and without MS at baseline were compared regarding development of new CKD (decreased eGFR). After exclusion of individuals with hypertension at baseline (*n* = 798), MS was not a risk for developed decreased eGFR (OR = 0.925, 95% CI; 0.446–1.917; *P* = 0.844). In an American Indian prospective cohort study [18], the adjusted hazard ratio metabolic syndrome was for eGFR less than 60 mL/min/1.73 m^2^ was 1.3 (95% CI, 1.0 to 1.6). The relationship between metabolic syndrome and kidney outcomes was stronger in those who developed diabetes during follow-up. One reasonable interpretation is that in the early phase of metabolic disorders related kidney disorders such as diabetic nephrology, GFR increases because of glomerular hyperfiltration.

In this study, we also compared the relationship between different criteria of obesity and CKD. Obesity diagnosed by waist circumference was associated with a higher risk for CKD. Body mass index in Chinese population was suggested to be different from WHO criteria [28, 29]. Higher prevalence of MS was reported despite lower BMI in Asian Americans [30]. In this study, we therefore defined obesity (BMI criteria) as BMI > = 25Kg/m^2^. In a previous Chinese study[31], association of CKD and anthropometric indexes (BMI, WC, etc.) were studied. Central obesity indexes (including WC) were concluded to be better than BMI for the prediction of CKD, especially in the blood-pressure-adjusted model. One cohort study suggest that the waist-to-hip ratio (WHR), but not BMI, is associated with incident CKD and mortality [32]. In our study, a different relationship between obesity using different criteria and the presence of CKD was identified. Obesity diagnosed by waist circumference or BMI was associated with a higher prevalence of CKD in age-and-sex-adjusted model, but after adjusting for hypertension and diabetes, the significance for obesity with BMI criteria disappeared. This may in part be because of the limitations of BMI in that it is unable to distinguish between weight from muscle and fat, between visceral and subcutaneous fat, and between peripheral and central adiposity.

There are several limitations in our study. First, a single measurement of urine and blood test was used to confirm the indicators of CKD and MS; therefore the reported prevalence of CKD and MS might be overestimated. Secondly, the definition of hypertension and diabetes was partly based on self-reported history and oral glucose-tolerance test was not done to confirm the diagnosis of diabetes. Finally, the cross-sectional design makes it difficult to infer causality between the MS and risk of CKD.

## Conclusions

In conclusion, our study shows that MS is prevalent in China; and its components is associated with a higher prevalence of CKD even after adjusting for hypertension and diabetes, which may mainly be contributed by the increased risk for albuminuria than that for decreased eGFR. Obesity diagnosed by waist circumference is associated with a higher prevalence of CKD than that diagnosed by increased body mass index. More attention must be paid to the population with MS, especially to that section of the population without diabetes or hypertension. People with elevated blood pressure and serum glucose are also the target population to receive albuminuria screening.

## Supporting Information

S1 Table Prevalence of MS and MS components. (DOC)

S2 Table Prevalence of CKD/CKD components with presence/absence of MS/MS components. (DOCX)
