# Supplementary material for: Metabolic Syndrome without Diabetes or Hypertension Still Necessitates Early Screening for Chronic Kidney Disease: Information from a Chinese National Cross-Sectional Study
Source: PLoS One. 2015 Jul 10;10(7):e0132220. doi: 10.1371/journal.pone.0132220 (PMC4498807; doi:10.1371/journal.pone.0132220)
Supplement: S1 Table — (DOC) [file pone.0132220.s001.doc]

***S1 Table.*** Prevalence of MS and MS components

| **Components number of MetS** | Male  ( %) | Female  (%) | Total  (%) |
| --- | --- | --- | --- |
| 0 | 29.26 (27.82-30.70) | 21.47 (20.21-22.72) | 25.42 (24.45-26.38) |
| 1 | 31.32 (29.91-32.73) | 27.50 (26.17-28.83) | 29.43 (28.47-30.41) |
| 2 | 22.22 (21.00-23.44) | 25.09 (23.81-26.36) | 23.63 (22.74-24.52) |
| 3 | 11.46 (10.55-12.38) | 15.75 (14.73-16.78) | 13.58 (12.89-14.26) |
| 4 | 4.73 (4.12-5.34) | 7.67 (6.95-8.39) | 6.18 (5.71-6.65) |
| 5 | 1.01 (0.73-1.29) | 2.52 (2.10-2.94)* | 1.75 (1.50-2.00) |
| MS (ATP-Ⅲ) | 8.65 (7.85-9.45) | 14.97 (13.99-15.95)* | 11.77 (11.13-12.40) |
| MS (ATP-Ⅲ modified) | 17.20 (16.11-18.29) | 25.94 (24.71-27.18)* | 21.51 (20.69-22.34) |
| MS(IDF) | 11.63 (10.70-12.56) | 21.86 (20.70-23.01)* | 16.67 (15.92-17.42) |

*: *P*<0.05, compared with male group. Abbreviations: MS=metabolic syndrome
