# Supplementary material for: Metabolic Syndrome without Diabetes or Hypertension Still Necessitates Early Screening for Chronic Kidney Disease: Information from a Chinese National Cross-Sectional Study
Source: PLoS One. 2015 Jul 10;10(7):e0132220. doi: 10.1371/journal.pone.0132220 (PMC4498807; doi:10.1371/journal.pone.0132220)
Supplement: S2 Table — (DOCX) [file pone.0132220.s002.docx]

***S2 Table. Prevalence of CKD/CKD components with presence/absence of MS/MS components***

| Croups | Decreased eGFR (%) | Albuminuria (%) | CKD (%) |
| --- | --- | --- | --- |
| Central OB1 | | | |
| No | 1.65 (1.41-1.88) | 10.06 (9.37-10.75) | 11.34 (10.62-12.05) |
| Yes | 2.55 (1.72-3.38) | 18.47 (15.98-20.95) | 20.05 (17.52-22.58) |
| Central OB2 |  |  |  |
| No | 1.53 (1.28-1.79) | 9.18 (8.41-9.95) | 10.38 (9.59-11.18) |
| Yes | 2.16 (1.72-2.60) | 14.47 (13.16-15.77) | 16.00 (14.66-17.34) |
| OB (BMI) | | | |
| No | 1.73 (1.46-2.00) | 9.82 (9.03-10.61) | 11.14 (10.32-11.96) |
| Yes | 1.76 (1.36-2.16) | 13.47 (12.20-14.74) | 14.76 (13.46-16.06) |
| High BP | | | |
| No | 1.29 (1.02-1.56) | 6.89 (6.10-7.69) | 8.00 (7.18-8.82) |
| Yes | 2.34 (1.96-2.72) | 16.36 (15.21-17.51) | 17.94 (16.77-19.12) |
| High TG | | | |
| No | 1.45 (1.22-1.69) | 10.28 (9.54-11.030 | 11.35 (10.59-12.11) |
| Yes | 2.74 (2.13-3.35) | 13.11 (11.56-14.66) | 15.28 (13.67-16.89) |
| Low HDL-C | | | |
| No | 1.54 (1.29-1.78) | 9.94 (9.16-10.72) | 11.15 (10.34-11.95) |
| Yes | 2.24 (1.75-2.73) | 13.31 (11.99-14.63) | 14.88 (13.51-16.24) |
| High FPG | | | |
| No | 1.46 (1.23-1.69) | 8.96 (8.26-9.67) | 10.14 (9.41-10.87) |
| Yes | 2.80 (2.18-3.43) | 18.37 (16.60-20.13) | 20.18 (18.38-21.99) |
| MS (ATP-Ⅲ) | | | |
| No | 1.50 (1.28-1.73) | 9.48 (8.79-10.17) | 10.64 (9.93-11.35) |
| Yes | 3.49 (2.60-4.38) | 21.60 (19.20-24.00) | 24.03 (21.57-26.49) |
| MS (ATP-Ⅲ modified) | | | |
| No | 1.28 (1.06-1.49) | 8.73 (8.02-9.44) | 9.76 (9.03-10.49) |
| Yes | 3.41 (2.74-4.09) | 18.84 (17.13-20.56) | 21.18 (19.42-22.95) |
| MS (IDF) | | | |
| No | 1.46 (1.24-1.69) | 9.34 (8.63-10.05) | 10.49 (9.76-11.21) |
| Yes | 3.10 (2.36-3.84) | 18.72 (16.79-20.66) | 20.84 (18.86-22.83) |

Central OB1: obesity according to WC by ATP-Ⅲ; Central OB2: obesity according to WC by ATP-Ⅲ modified or IDF; Obesity (BMI): >=25Kg/m^2^

Abbreviations: BP=blood pressure; FPG=fasting plasma glucose; HDL-C=high density lipoprotein cholesterol; OB=obesity; BMI=body mass index;
